# Transient multimers modulate conformer abundances of prion protein monomer through conformational selection

**DOI:** 10.1038/s41598-019-48377-w

**Published:** 2019-08-21

**Authors:** Guillaume Van der Rest, Human Rezaei, Frédéric Halgand

**Affiliations:** 1 Université Paris Sud-CNRS, Laboratoire de Chimie Physique, Bâtiment 201 P2, F-91405 Orsay, France; 2 Institut National de la Recherche Agronomique, UMR 892, Virologie Immunologie Moléculaires, Domaine de Vilvert, F-78350 Jouy-en-Josas, France

**Keywords:** Intrinsically disordered proteins, Predictive markers

## Abstract

Prions are known to be involved in neurodegenerative pathologies such as Creutzfeld-Jakob disease. Current models point to a molecular event which rely on a transmissible structural change that leads to the production of β-sheet-rich prion conformer (PrP^Sc^). PrP^Sc^ itself has the capability to trigger the structural rearrangement of the ubiquitously present prion (PrP^c^) substrate in a self-perpetuating cascade. In this article, we demonstrate that recombinant PrP^c^ exists in a conformational equilibrium. The conformers’ abundances were shown to be dependent on PrP^c^ concentration through the formation of transient multimers leading to conformational selection. The study of PrP^c^ mutants that follow dedicated oligomerization pathways demonstrated that the conformers’ relative abundances are modified, thus reinforcing the assertion that the nature of conformers’ interactions orient the oligomerization pathways. Further this result can be viewed as the “signature” of an aborted oligomerization process. This discovery sheds a new light on the possible origin of prion protein diseases, namely that a change in prion protein structure could be transmitted through the formation of transient multimers having different conformer compositions. This could explain the selection of a transient multimeric type that could be viewed as the precursor of PrP^Sc^ responsible for structural information transmission, and strain apparition.

## Introduction

Only a few analytical methods, such as NMR^1^ or molecular dynamic simulation^2^ allow the study of the conformational landscape of proteins^1,2^. An alternative to these techniques relies on “native” electrospray ionization mass spectrometry that has become a valuable tool for the study of protein assemblies in the past years^3^. More recently, implementation of ion mobility in commercial mass spectrometry instruments has led to a renewed interest for this technology in the field of structural biology. From now on ion mobility is a method of choice to describe the native conformational properties of proteins^4–6^. This approach allowed the study of Syrian (golden) hamster prion protein (PrP) conformers^7^ and more recently our group deeply described the ovine prion protein structural features^8^.

Prion protein is known to be involved in neurodegenerative pathologies such as Creutzfeld-Jakob disease in humans. Current models responsible for such pathologies rely on a transmissible conformational change that leads to the production of an amyloidogenic β-sheet-rich prion called PrP^Sc^ ^9,10^. While the ability of PrP^Sc^ to form amyloid fibrils was widely studied previously^11,12^, the formation of highly cytotoxic, beta-sheet rich oligomeric structures^13^ still remains elusive. Transient oligomeric assemblies could constitute an important object of interest for their implication in the conversion of healthy Prion protein (PrP^c^) into the pathological PrP^Sc^ conformer. It was recently reported that PrP^Sc^ assemblies, from different prion strains, combine into highly stable and oligomeric elementary building blocks called suPrP, that harbour the prion protein strain structural determinant^14^. In physiological conditions a highly dynamic equilibrium between suPrP and PrP^Sc^ exists undermining the widespread idea of rigid and deadpan amyloid structuration of PrP^Sc^ assemblies. The demonstration of the presence of oligomeric elementary building blocks questions the molecular mechanisms of the templating process and how these oligomeric elementary building blocks intervene in the templating process leading to the growth of PrP^Sc^ assemblies.

During other work on PrP behavior, we incidentally observed changes in charge state distributions (CSD) along a chromatographic peak. This observation sparked our interest, as this observation could be due to the evolution in concentration along the eluting peak, and that it was already shown that prion neurotoxicity could be related to alternate PrP^Sc^ isoforms whose production is concentration dependent^15^. Thus, we hypothesized that some selected conformers of monomeric PrP^c^ could self-assemble to form transient, short-lived, oligomeric species in a concentration-dependent manner, thus disrupting the equilibrium between conformers that we and others had previously observed^7,8^. This article will describe our initial observations as well as other “native” mass spectrometry experiments that confirm this hypothesis and demonstrate this effect both for wild-type (*wt*) Prion protein (PrP) monomer as for some mutants which have specific oligomerization pathways. To that goal we performed “native” mass spectrometry experiments coupled with ion mobility to probe protein concentration related conformational changes. Our data demonstrate that protein concentration affect the conformer family abundances for wild-type (*wt*) Prion protein monomer as well as for mutants leading to specific oligomerization pathways.

## Material and Methods

### Material

Horse heart myoglobin and *Saccharomyces cerevisiae* alcohol dehydrogenase were purchased from Sigma Aldrich. Triethylammonium acetate (TEAA) solution (1 M, pH 7) at the highest purity grade (BioUltra), was obtained from Fluka.

### Methods

#### Production of wt and mutant PrP

The full-length ovine PrP^C^ (Ala 136, Arg 154, Gln 171 variant named ARQ) was expressed in *E*. *coli* and purified as described previously^16^. Mutants (H190A, I208M and I206A) as well as N-terminal and C-terminal constructs were prepared by introducing the point mutation at the desired position or specific plasmid for constructs and expressed and purified as for the ARQ protein. Mutant H190A, was chosen because it is the only amino acid that maintain the β-strand-S1/α-helix H1 and β-strand S2 to α-helices H2 and H3 bridge by a disulfide bond making the core of the globular part (C-terminus) of the PrP^c^. Consequently, His 190 to Alanine mutation leads to the disruption of this salt bridge and induces a destabilization of the structure. Mutant I208M was chosen based on sequence differences between mouse and sheep, and knowing it impairs oligomer production, yielding O3 oligomer only^11^. Finally, I206A mutant was selected as a control since it doesn’t modify the formation of oligomers^11^. N-terminal and C-terminal constructs were built to define which domain predominantly affect conformational equilibrium. For the C-terminal construct, a His-tag was inserted for purification as no octarepeat regions are present to allow purification on Sepharose column loaded with copper. Insertions and substitutions in the primary sequence of ARQ were introduced by site-directed mutagenesis (QuikChange II mutagenesis kit, Stratagene) of ARQ cloned into pTRE plasmid (Clontech) using mutagenic primers^17^. The sequences of all the mutants were verified by sequencing. Numbers indicated for each insertion refer to the position of the first amino acid of the insert according to the sheep PrP^c^ numbering. Briefly, the gene encoding the full-length PrP was cloned in a pET 22b+ vector and expressed in the BL21 DE3 Escherichia coli strain after IPTG induction. The expressed PrP^c^ proteins accumulated in inclusion bodies. After lysis, sonication and solubilization of the inclusion bodies by guadinium chloride, purification of the prion protein was performed on a Ni Sepharose column loaded with copper. Refolding of the protein was achieved on the column by heterogeneous phase renaturation simultaneously to purification. The protein was recovered in triethylammonium acetate buffer (10 mM, pH 3.3) by elution on a G25 desalting column.

#### Preparation of protein solutions

*Wt* (ARQ) and, H190A, I208M, I206A mutants as well as N- and C-terminal constructs, were diluted at the appropriate concentration (0.05 to 80 µM) in a 20 mM triethylammonium acetate (TEAA) buffer at pH 3.35. Spectra were recorded from low to high protein concentrations to avoid carry over effects.

#### Mass spectrometry analyses

Mass spectrometry (MS) experiments were performed on a QToF instrument (Synapt G2-Si, Waters Company, Manchester, UK) equipped with a Traveling Wave Ion Mobility (TWIM) cell^18^. For direct infusion, samples were introduced in the ESI source at a flow rate of 5 µl/min and analyzed in positive ion mode over the *m/z* 500 to 5000 mass range under “native” conditions. Calibration was performed using sodium trifluoroacetate. The typical standard error was below 5 ppm. Reliable and accurate detection of native proteins was obtained by optimizing instrumental and biochemical parameters as described in a previous work^19^ and in agreement with the work by Chen and Russell^20^. For “native” experiments instrumental and hardware parameters were optimized to reach the best average signal to noise ratio while preserving intact quaternary and tertiary structures. Ion mobility data were also recorded in the same experiments. For analysis under non-denaturing conditions the following instrumental parameters were used: capillary voltage = 4.5 kV, sampling cone = 150 V, source offset, 150 V, nebulization gas pressure = 5 bar, source temperature, 40 °C, desolvation temperature, 75 °C. For ion mobility (IMS) experiments in nitrogen as drift gas, the following instrumental parameters were used:: Trap gas flow = 7 ml/min; He gas flow = 120 ml/min, IMS gas flow = 45 ml/min, Trap wave velocity = 300 m/s, Trap wave height = 4 V, IMS wave velocity = 800 m/s, IMS wave height = 40 V, Transfer wave velocity = 110 m/s and Transfer wave height = 4 V.

#### Data analyses, processing and interpretation of ion mobility experiments

After recording ion mobility data, we extracted all information, such as mass to charge ratios (*m/z*), drift times (*t*_*d*_) and intensities, using the peak detection procedure of Driftscope^TM^ software. All the data were gathered in a raw data file (.csv). Then our home-made script^8^ allowed extracting information only related to the protein of interest by entering its average molecular mass, the *m/z* accuracy, limits of the charge states distribution as well as collisional cross section (Ω = CCS = collisional cross sections) calibration parameters. This allowed us to rapidly process the data and plot Ω = f (z) (Ω = CCS = collisional cross sections; z = charge state) graphs to reveal the conformational landscape of PrP that were used to explore our data. In Table S1, drift times for each charge states are displayed and allow associating single and overlapping conformer families. In a quantitative point of view, for each charge state and each protein concentration of each ovine PrP, intensities at the peak apex of the mobilogram were automatically extracted and saved in an Excel sheet. Summation of maximum ion mobility peak intensities for the specific drift time and charge state of each conformer family, allowed calculating the relative intensities (abundances) of each conformer family for each protein concentration. For detailed information see Supporting information.

## Results

### First observation

**Figure 1:**
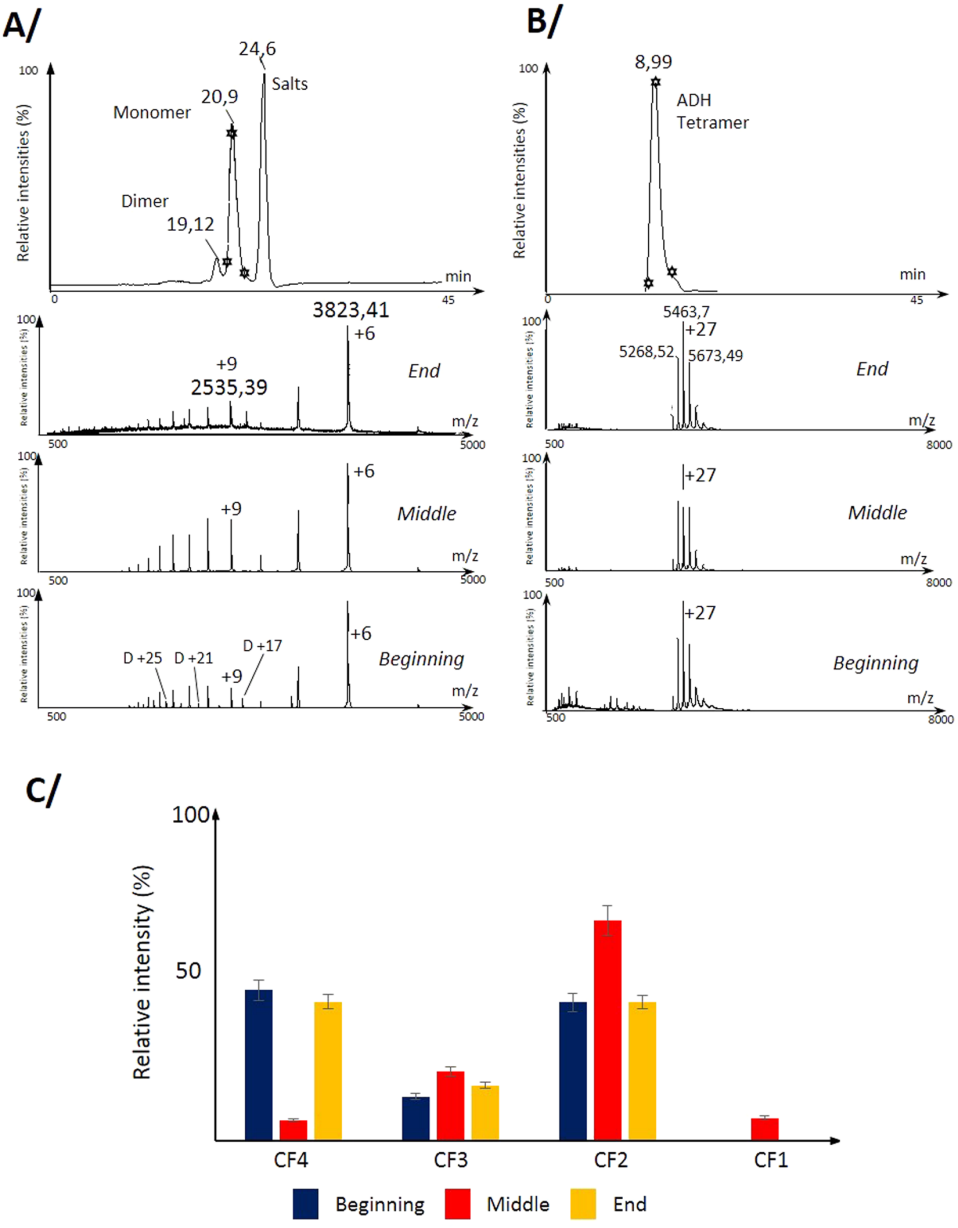
First evidence of an effect of protein concentration on the conformer intensity ratios. (**A**) Observation of changes in charge state distribution (CSD) reflecting, protein protonation propensity or in other words accessibility of protonable residues in the protein, under the monomer chromatographic peak for the I208M PrP mutant (22934 Da) at the front, top and end of the chromatographic peak (See stars). (**B**) Control experiment using the alcohol dehydrogenase tetramer (ADH, 147 493 Da) in identical buffer, pH and SEC conditions showing no change in CSD. (**C**) Bar graph displaying the relative intensity ratios calculated based on ion-mobility data of conformer families for the H190A mutant within the monomeric SEC chromatographic peak. The variation of the CSD as a function of protein concentration suggests the existence of different Prion protein structures.

Considering our observations regarding the existence of multiple folding pathways^8,19^ of PrP, we used monomeric PrP and mutants to explore the dynamics of the conformational landscape of the protein. As shown in Fig. 1A, we observed that in size-exclusion chromatography (SEC) coupled to ion mobility and mass spectrometry (IMS-MS), the charge state distribution (CSD) of I208M mutant recorded under “native” conditions evolves within the chromatographic peak. Under these conditions the bimodal CSD (+5 - +7 and +8 - +16) shows changes in their respective charge states (CS) intensities with the observation of an increase in the (+8 - +16) CSD at the middle of the chromatographic peak while (+5 - +7) CSD is predominant on the front and tail edges. Interestingly, this phenomenon which is observed for all PrP’s (*wt* and mutants) was not observed for the dimer separated by SEC chromatography (see Fig. S2). But on the other hand, one could argue that dimer concentrations at the front and tail edges being much lower than monomer, the threshold might not be reached even at the top of the chromatographic peak. Litterature has shown that charge state distribution can be used as an indicator of the folded nature of a protein^8,21^, thus this behavior could indicate a change in conformation in the course of the chromatographic peak elution. The variation of the CSD suggests the existence of different structures, which can be observed using ion mobility, and quantified into conformer families by our methodology as described previously^8^ (See Fig. S1 for an example on ARQ conformational landscape). Changes in relative intensities ratios of conformer families (CF) are observed along the chromatographic peak (Fig. 1C). To exclude the hypothesis that this phenomenon could be due to the chromatographic separation or electrospray (ESI) process, alcohol dehydrogenase tetramer (ADH) was used as a control under identical SEC column, protein concentration, and buffer and pH conditions. Data obtained showed no change in either in CSD nor in ion mobility profile for different areas of the chromatographic peak (Fig. 1B). These observations confirm a structural origin of these changes and demonstrate that they are not generic to all proteins. We thus hypothesized that the parameter mostly changing along the chromatographic peak was protein concentration and validated this hypothesis by direct infusion of PrP solutions of known concentrations.

### Probing conformer abundance modulation by protein concentration

**Figure 2:**
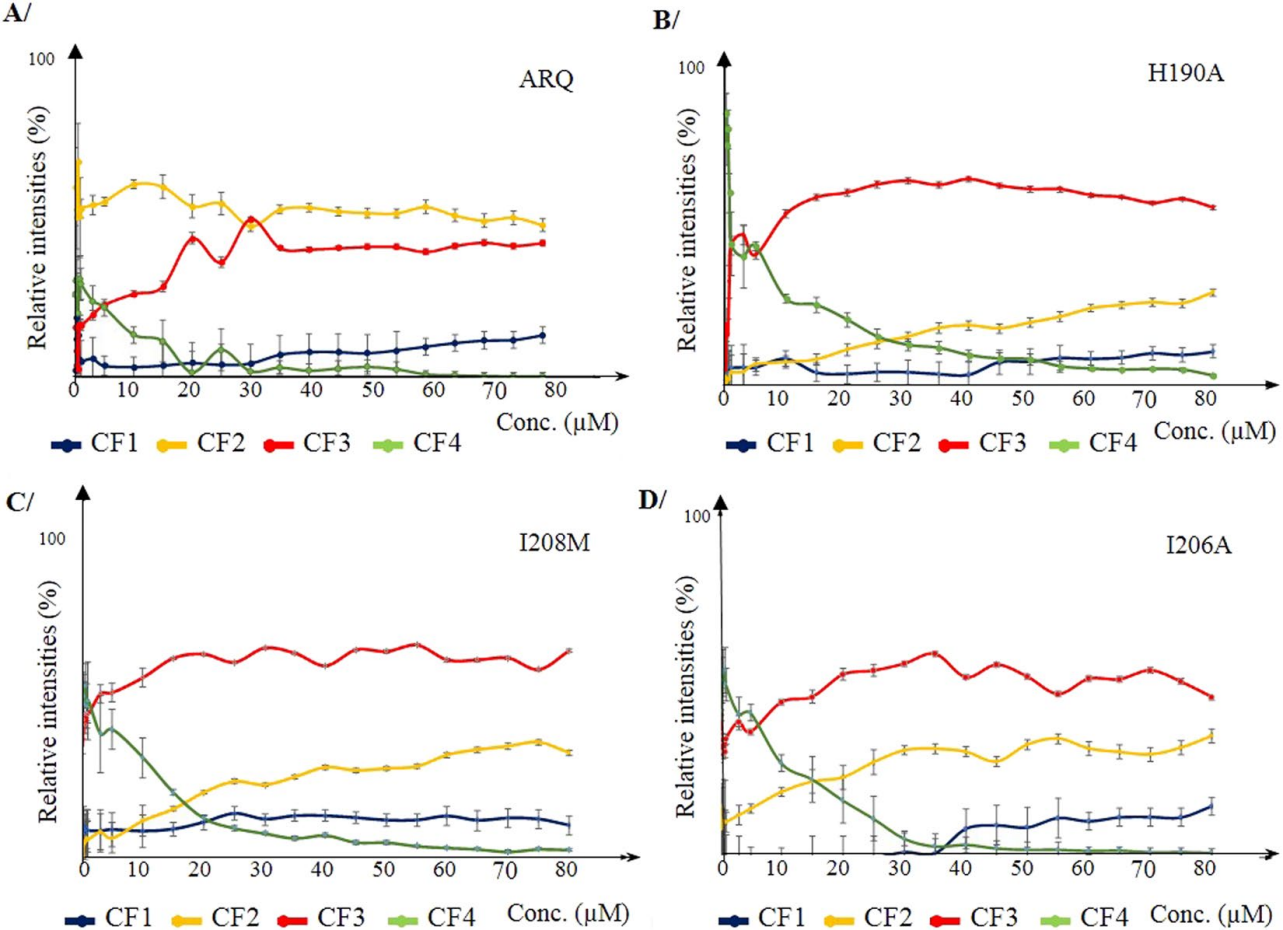
Protein concentration effect on conformation family abundances for PrP ARQ and mutants. Plot of the relative intensities of the conformer families of prion proteins based on ion mobility data: (**A**) ARQ (*wt* PrP); (**B**) H190A, (**C**) I208M and (**D**) I206A mutants in 10 mM TEAA pH 3.3 under “native” conditions as a function of protein concentration (0.05 to 80 µM). Mutants display a different conformer family profiles with respect to the *wt* PrP^c^ with increasing protein concentration. In contrast, mutants share similar evolution of the conformer intensity ratios between each other and only mainly differ by their respective intensity ratios at a given protein concentration. This demonstrates that mutations alter structural and conformational equilibrium with respect to protein concentration.

With the view to demonstrate that, according to our initial observation, protein concentration modulates conformer family abundances through structural changes we recorded ion mobility and mass spectrometry data for protein concentrations ranging from 0.05 to 80 µM under direct infusion in ESI source. Data confirmed that conformer abundances vary with protein concentration (See Fig. 2A). For ARQ one can observe that conformer CF4 family intensity strongly decreased as concentration increase from 0 to 20 µM and reached a minimum value (<2%) at 80 µM. In the meantime, conformers CF1 and CF3 abundances were found to regularly increase with CF3 and CF1 reaching about 45 and 5% intensity, respectively. Finally, intensity ratio of conformer CF2 slightly decreased with increasing concentration. These results demonstrate that conformer family’s abundances are modulated by protein concentration. A recurrent concern when conducting gas phase experiments to probe protein structures is that the observed gas phase structures might not reflect actual changes in solution. In our experiments the only parameter that was changing was the protein concentration, which means that changes in relative gas phase conformers’ abundances are directly correlated to solution phase changes. We conclude that the effect of protein concentration upon ARQ structures in solution is reflected in the gas phase. One interrogation here refers to whether it is possible to provide insights into the structures of the conformers. In the state of the art of ion mobility experiments the only accessible information is the collisional cross section that denotes the shape of a conformer. Here, ion mobility data are recorded during a 22 msec time scale while possible changes in structure are thought to occur after 30 msec^22^, suggesting that no structural change likely occurred in the course of the IMS experiment. No additional structural information can be obtained. However, this could be related to the calculated surface of ARQ (Ω in Å2), based on the assumption that ARQ could be described as a stoke particle having a hydration sphere surface of 3846 Å2. A value in the same order of magnitude than CCS (Ω in Å2) of conformer families ranging from 1000 to 2500 Å2 ^8^. We also tried to use hydrogen to deuterium exchange to probe the structures of the conformers and gain insights on structure accessibilities. Unfortunately, conformers interconvert very rapidly, and the deuterium content of each conformer is the same, since it corresponds to an average of deuterium exchanges of all conformers (data not shown).

### The N- and C-terminal domains of ARQ contribution to the conformational landscape

**Figure 3:**
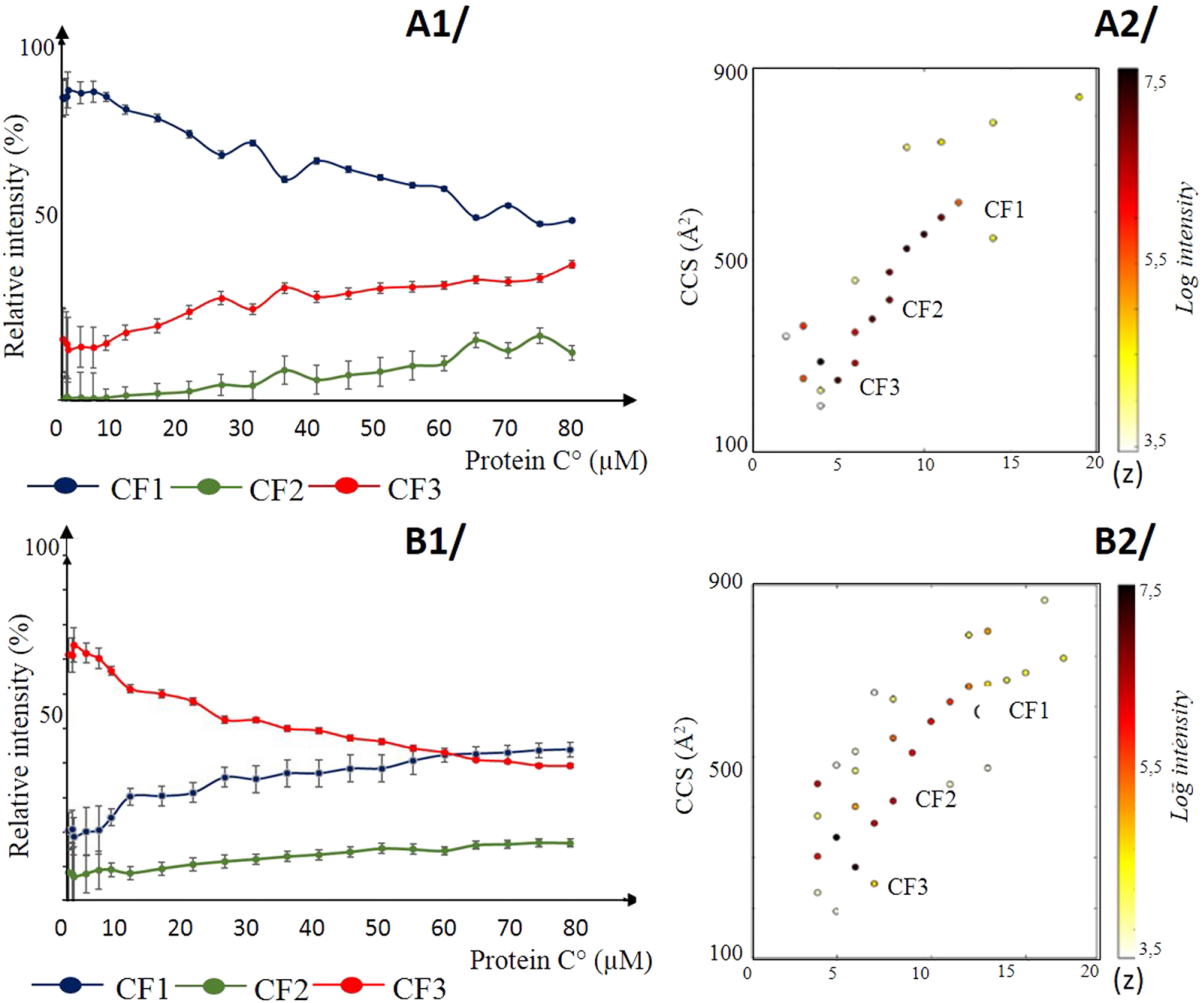
Exploration of the effect of protein concentration on conformational landscape for N-ter and C-Ter constructs. Data related to (**A**) ARQ N-ter, and (**B**) C-ter domain bearing a His-Tag. Figure A1 and B1 are plots of the intensity ratios evolution of conformer families (CF1 to CF3) for increasing protein concentration. Figure A2 and B2 correspond to conformational landscapes for N-ter and C-ter constructs. Dots refer to collisional cross sections (CCS Å^2^) for each charge state. Lines denotes conformational families (CF1 blue, CF2 green and CF3 red).

To determine if either the unfolded N-terminal domain (ARQ N-ter = 23 to 103 AA with MW_av_ of 9960 Da) and/or the C-terminal globular part which bears a His-tag (ARQ C-ter = 124 to 234 sequence having an MW_av_ of 16167.7 Da, are involved in the concentration based structural changes, we used the same procedure to separately look at the conformational landscapes of these two constructs. We postulated here that the His-tag carried by the C-terminal part should not significantly alter its structural properties. However, we point out that results obtained for the C-terminal construct must be taken with caution since the presence of a His-tag could both modify the charge state distribution and alter the structure of this domain. Conformational landscape plotting (Fig. 3–1B and 3–2B) of each domain reveals that they are both represented by three conformer families, labeled CF1 to CF3 from the more open structure to the most compact one. Plotting the relative abundances of each conformer family with respect to protein concentration demonstrates that both domains seem to be involved in the Prion protein conformational landscape modulation. This is exemplified with the observation of three conformers that displayed a high plasticity whatever are the properties of each domain. Indeed if N-terminal domain that is known to be unstructured is expected to be highly dynamic, in case of the C-terminal globular domain that is expected to be compact and “rigid” conclusions were hard to draw. (Fig. 3–1B and 3–2B).

### Effect of mutations on the conformational modulation

To determine if the concentration dependence conformational landscape of ARQ could be affected by specific mutations that modify the heat induced oligomerization pathways, we identically probed the effect of protein concentration for each mutant. Thus, mutants H190A and I208M that lead to the production of O1 and O3 oligomers^11^, respectively, and mutant I206A that does not modify the oligomerization pattern compared to *wt* ARQ were studied. First, it is noticeable that for all mutants studied, conformer’s abundance profiles were similar and only differed by their relative intensities (Fig. 2B–D). Nevertheless, small differences were observed between mutants such as for CF1. As expected from previous work^8^, the ARQ conformational profile was found clearly different from mutants. To conclude, these results demonstrate that whatever is the nature and the extent of the structural perturbation, mutations endorse similar changes in conformational landscape but with different degrees for each conformer in comparison with ARQ. Finally, another result of importance relates to the fact that no non-specific aggregation forms (trimer, tetramer, …) were found, which confirms that non-specific aggregation is not at the origin of the changes observed in conformational profiles.

Finally, detection of a dimer in the mass spectra of ARQ and mutants raises the question of the existence of an equilibrium between the dimer and monomeric ARQ and mutants. Relative abundances of the dimers were plotted as a function of protein concentration. Note that dimer amount is underestimated since only odd peak intensities could be considered due to overlapping of even dimer CS with monomer peaks in the mass spectra. Yet, by rigorously using the same calculation procedure, the dimer amount was found to be independent from the protein concentration (Fig. S3 of supporting information). A likely explanation is then that the dimer is an expression or production artefact of PrP^c^ and has no relevant role in the conformational profile of the monomer.

#### Validation of our results

With the view to ensure that CSD (charge state distribution) and related conformational changes observed for PrP were reliable, myoglobin was used as a standard to probe variation of CF (conformer families) ratio intensities in ion mobility under identical conditions. Mass spectra revealed that myoglobin is represented by a bimodal distribution thus suggesting, in agreement with literature^23^, the presence of at least two conformations in the gas phase (See Fig. S3). By plotting the relative intensities of the respective conformer family with respect to protein concentration we can observe that ratios are rather constant with small variations for lower (Δ 0.6%) and higher (Δ 5.6%) protein concentrations. This was also tested for apo myoglobin conformers, showing that the protein concentration had no effect on the conformer relative abundances^23,24^ (See Fig. S4 of supporting information). Under these conditions reproducibility was calculated from all charge state intensity of respective myoglobin conformer and for triplicate injections day to day, giving a standard deviation (σ) of 3.1%. This result allowed us to conclude that conformer variations recorded for prion protein under different protein concentrations are significant. Consequently, this means that changes in CF ratios reflect, at least partially, solution behavior, even if calculated ratio intensities do not necessarily reflect solution equilibrium. To further substantiate our methodology, we determined the reproducibility of our experiment. Analyses were performed for all PrP proteins and for different concentrations in triplicate, and for three different protein solutions. As expected, standard deviations were much higher at low concentration (e.g. for CF4 at 0.5 µM σ was 6.5%) than at high concentration (e.g. for CF2 at 60 µM σ was 1.2%) and varied with CF. Error bars in graphs were added with respect to standard deviations calculated for each conformer families. See Table S2 of supporting material. Finally, we assessed that chemical noise did not impact CF ratio intensities determination. Consequently, we neglected the contribution of this factor.

## Discussion

The existence of multiple conformers of PrP is the basis for selection of the best substrate for replication, host adaptation and prion protein evolution^25–27^. Our study shows that α-helix rich form of ARQ and mutants monomer conformer ratios are modulated by concentration changes. In addition, the recent detection of highly stable and oligomeric elementary building blocks called suPrP harboring the PrP strain structural determinant^14^ questioned the role of conformer families in the PrP^c^ to PrP^Sc^ conversion and in the templating process. The actual dogma considers that the primary structure of PrP governs its structural landscape and therefore its conversion to PrP^Sc^ as well as PrP^Sc^ transmission^28^. The fact that the concentration of the monomeric PrP^c^ affects its structural landscape adds another dimension to the PrP^c^ landscape modulation. Indeed, depending on cells or tissue level of expression, the PrP^c^ landscape could vary and consequently evolve toward a PrP^Sc^ strain that present specific cell/tissue tropism^29^.

Whatever the details of CFs evolution, the first evidence is that the protein concentration governs these changes. The corollary of this assumption is that modulation of CFs profiles is dependent on an increase in interactions that are believed to occur transiently between different monomeric PrP^c^ molecules. According to our observations the changes in CF abundances are similar in static concentration conditions (direct infusion) compared to dynamic conditions (chromatographic separation). Thus, displacement in conformation equilibrium occurs at a speed comparable with that at the basis of the chromatographic separation (lifetimes below 1 minute). Since, all mutants displayed the same trend in conformational landscape modulation with protein concentration increase it is, at that stage, impossible to point out which conformers are responsible for orienting the oligomerization pathways. This can be resolve by the fact that these conformers can be viewed as “intermediates” in conformational landscape changes or toward enrichment of one CF with respect to protein concentration changes. However, this is in apparent conflict with SEC data. Indeed, such a phenomenon could arise from the formation of transient multimers, however we never observed these last or a shift of the SEC peak toward lower retention times. One explanation could be that these multimeric species would be present at very low concentration. In this case, the infinite dilution linked to the dynamic elution process of the multimers in SEC could impair their detection^30–32^. Nonetheless, the detection of different PrP^c^ conformational landscapes implies that transient multimers are thermodynamically favored. Such phenomenon could only occur via a conformational selection process a concept that is the cornerstone of the prion protein theory^33^. This is in full agreement with previous works demonstrating that a sequential two step multimolecular process occur for oligomer formation and their ratio partition, both depending on the initial protein concentration^11^. Similarly, mutants’ conformational landscape evolution involves conformational selection processes. However, in these cases if mutants were shown to follow the same trend, the nature of the transient multimers were thought to differ from one mutant to another. Consequently, the composition of the transient multimers were thought to be divergent between mutants themselves and of course from PrP^c^. Following this statement this could then explain the origin of the plurality of oligomerization pathways. Considering, N- and C-terminal domains CF evolution with protein concentration, information obtained demonstrate that N- and probably C-ter domains play a role in PrP^c^ plasticity and dynamic.

**Figure 4:**
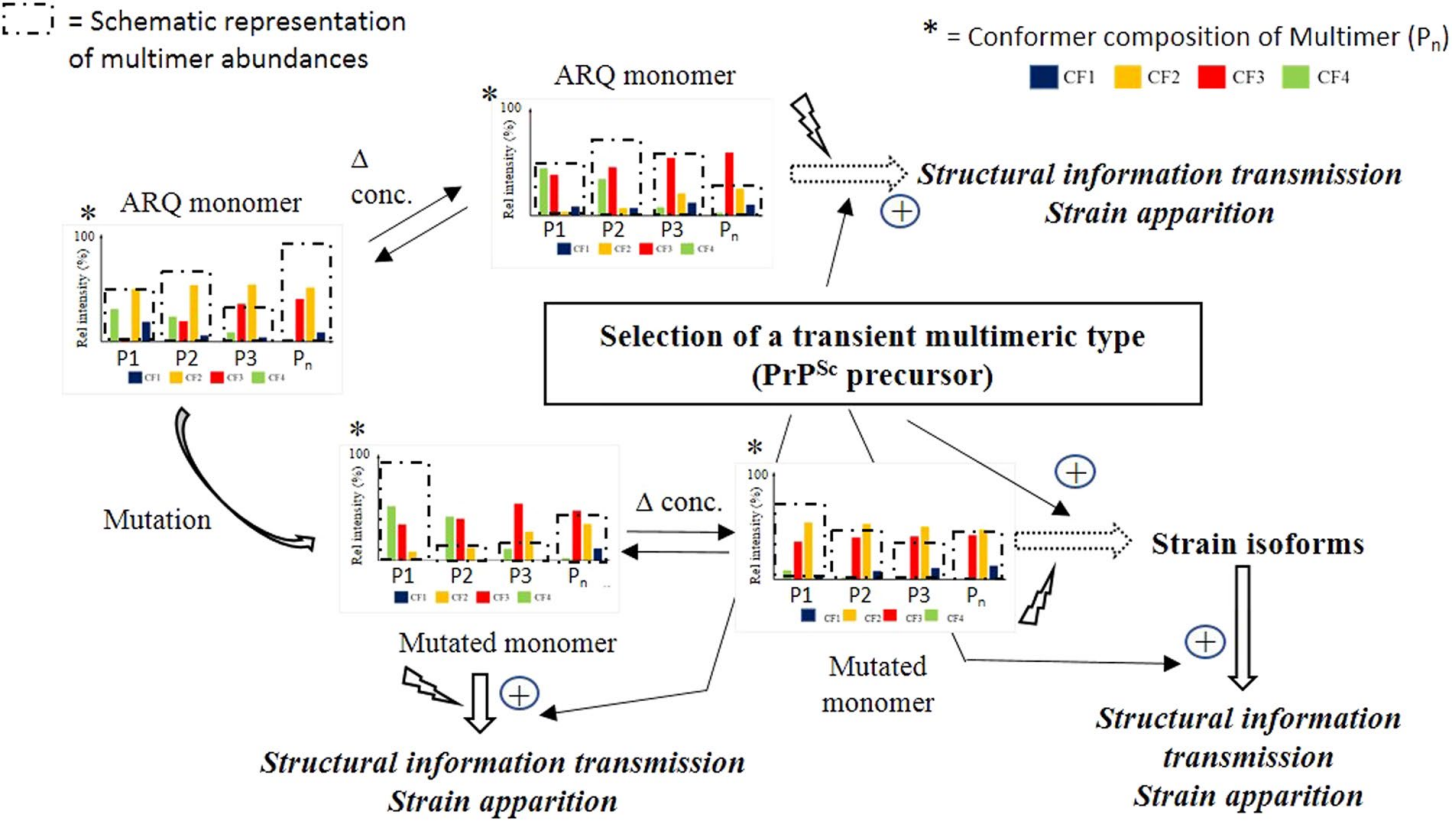
Proposed scheme illustrating the modulation of conformer ratio intensities of PrP monomer with respect to protein concentration or mutation. In this case changes in conformer intensity ratios involve the conformational selection via the formation of transient multimers or by changing the relative abundances of transient multimers having stable conformational profiles. These transient multimers exchange structural information then modifying the conformational pattern of monomers. Factors affecting transient species profiles are the occurrence of mutation and protein concentration changes but could also be link to the respective abundances of the different multimers having stable conformer abundances inside multimers. The selection of a transient multimer is viewed as the precursor of pathological Prion protein. Thus, such precursor could explain strain apparition and transmission. *Legends*: CF refer to conformer families (CF1 = blue, CF2 = yellow, CF3 = red and CF4 = green) making up the transient multimers (P_n_); Boxes with dash lines represent the relative abundances of transient multimers; Δ conc. = protein concentration variations.

To summarize our results (Fig. 4) we first need to define structural levels that are described in Fig. 4. Thus, the first level concerns the initial monomer that is represented by four conformers. The second level is represented by transient multimers that contain several monomers presenting similar or different conformer ratios. The third level refers to the respective intensity ratios of multimers having stable conformer compositions. Then through conformational selection one or several conformers generate new conformational patterns then orienting the nature (conformational landscape) of the monomers making up the transient multimers. Factors affecting transient species profiles are the occurrence of mutation and protein concentration changes but could also be linked to the respective abundances of the different multimers (dashed boxes). As stated before, these multimers are transient and dissociate quickly into monomers. But monomers could keep the conformational pattern they acquired in multimers. This last structural level is the key point where prion protein could evolve toward a PrP^c*^ precursor that will be the entry point for oligomerization / fibrillation. Then factors such as mutation or a change in protein concentration, that affect prion protein conformational equilibrium could promote the apparition of the PrP^Sc^ precursors that will evolve or not toward a pathological object. Such process is reversible and when protein concentration decreases conformational landscape return to initial equilibrium. These results could be related to the appearance of new PrP^Sc^ strains *in vivo*^34^ but also to the self-perpetuation of the structural information stored in PrP^Sc^ involving suPrP highly dependent on PrP^c^ concentration^14^. In our case, this could be related to Prion protein conformer’s ratio differences, between the donor and recipient knowing that propagation proceeds mainly, when interacting PrP^c^ and PrP^c*^ are of identical structures, or based on our results on identical conformer’s composition^28^. This result could also explain multimers barriers, in agreement with early studies demonstrating that transmission barrier resides in the PrP primary structure differences dictating the PrP^Sc^ conformational landscape. In our case, this could be related to PrP^Sc^ conformer ratio differences, between the donor and recipient. Propagation then mainly proceeds when PrP^c^ intermediate conformer named PrP^c*^ and PrP^c^ of identical structures interact, or based on our results on identical conformer’s composition^28^. With respect to our results we can introduce another concept that would refer to the selection of a transient multimeric type (PrP^C*^) that could then be viewed as the precursor of PrP^Sc^ responsible for structural information transmission. Such hypothesis could explain strain apparition and selection. In addition since strains consist of an ensemble of molecular multimers containing a preferentially PrP^c^ then selected populous subspecies could promote a strain shift^28^. Such hypothesis agrees with the detection of molecular diversity in PrP isolates (Fig. 4).

## Conclusion

In this study, the coupling of “native” mass spectrometry with ion mobility allowed revealing a unique property of the prion protein monomer, namely the modulation of the conformer ratio intensities by protein concentration through the formation of transient intermediates. This discovery, that has not escaped our attention, sheds a new light on the possible origin of prion diseases, which would imply that change in PrP^c^ conformational landscape could be transmitted through the formation of transient multimers. Implication of transient multimers having different compositions in conformers could here explain the selection of a transient multimeric type and could then be viewed as the precursor of PrP^Sc^ responsible for structural information transmission, and strain apparition. In perspective, we are currently trying to characterize the nature and properties of these transient multimers.

## Supplementary information

Supporting information
