# Supplementary material for: Transient multimers modulate conformer abundances of prion protein monomer through conformational selection
Source: Sci Rep. 2019 Aug 21;9:12159. doi: 10.1038/s41598-019-48377-w (PMC6704068; doi:10.1038/s41598-019-48377-w)
Supplement: Supplementary file 1 — Supporting information [file 41598_2019_48377_MOESM1_ESM.docx]

**Transient multimers modulate conformer abundances of prion protein monomer through conformational selection.**

**Guillaume Van der Rest ^1^, Human Rezaei ^2^, Frédéric Halgand ^1 *^**

^1^ Université Paris Sud-CNRS, Laboratoire de Chimie Physique, Bâtiment 201 P2, F-91405 Orsay, France. ^2^ Institut National de la Recherche Agronomique, UMR 892, Virologie Immunologie Moléculaires, Domaine de Vilvert, F-78350, Jouy-en-Josas, France.

* Author for correspondence : Dr F. Halgand, Université Paris Sud- CNRS, UMR 8000, Rue Ampère, bâtiment 201 porte 2, F-91402, Orsay, France. Email: frederic.halgand@u-psud.fr; Phone : +33 1 69 15 82 51.

**Supporting information.**

***1/ Method for data analyses and processing of ion mobility experiments***

The procedure for data analysis of ion mobility experiments was the following. First ion mobility spectra were opened in Driftscope^TM^ software. Then we performed a peak detection using a resolution of 2000 and a signal threshold detection of 2000 counts. At this stage an Apex3D ionfile.csv is created. From this file, data (*m/z*, intensities, drift times, etc.) related to our protein were extracted using a home-made script. This script is accessible on request to the authors. From this script an Excel file was saved. Following this extraction, since it was shown by seminal work of Schvartsburg & Smith ^1^ that the drift time in TWIMS cannot be directly correlated to absolute collisional cross-sections (CCS = Ω) we processed data according to the protocol referring to CCS (Ω) calibration in TWIMS defined by Ruotolo, et al ^2^ in order to obtain an estimate of the absolute collisional cross section. In this procedure, it is first required to calibrate CCS. For that purpose, denatured myoglobin was used since Charge State Distribution (CSD) of this last globally cover the CSD of the PrP and that both proteins have roughly similar masses (16,9 kDa for myoglobin and 23 kDa for PrP). This calibration was performed with mobility parameters strictly identical to those used for measuring PrP. The *X* factor was determined by first calculating the drift time corrected for transfer time of flight using equation (1): $td^{'}=td-C\sqrt{\frac{m/z}{1000}}$, where C was extracted from the instrumental parameters as C = 1.41, followed by calculation of Ω’ that correspond to the cross section of the species independent from the charge and the nature of the gas using equation (2): $\Omega^{'}=\frac{\Omega}{z \sqrt{\mu}}$where µ correspond to $\mu= \frac{Mion*Mgas}{Mion+Mgas}$. Then ln (Ω’) = X ln (td’) + ln (A) is plotted. A linear regression is then applied to determine the X factor and ln A value. In our case and for myoglobin the linear regression equation was $y=0.6262 x-2.1507$ with a R^2^ of 0.9956. Thus, X has a value of 0.6262 and ln A is 2.1507. This X factor value was then used to calculate the final corrected values of the drift times with equation (3):$td"= {td'}^{X} (\frac{z}{\sqrt{\mu}})$. Finally, the $\Omega=a td" +b$equation was plotted for the reference compounds, and factors a and b were determined with respective values of a = 260.62 Å²/ms) and b = 110.89 Å^2^. Thus, after determination of these factors we calculated td” and Ω for all the protein studied under different conditions. Then $\Omega=f (z)$ was plotted for all protein and all experimental conditions and used to describe our data. It appears from our data that some points in the $\Omega=f (z)$ plots were shown to be “contaminations” that correspond to chemical noise and not to the proteins.

After recording the ion mobility data, we searched a way to merge the information obtained from ion mobility experiments with information known on the protein under study. To that purpose the peak detection procedure of Driftscope^TM^, that extracts all information related to the full experiment, was used to generate a raw data file containing all information (e.g. *m/z*, *t_d_*, intensities, CCS) for the peaks identified in the experiment. Then our home-made script allowed extracting information only related to the protein of interest by entering its average molecular mass, the *m/z* accuracy and limits of the charge states distribution based on the information extracted from MassLynx^TM^. This allowed us to rapidly process the data and plot Ω= f (z) graphs to reveal conformational landscape of PrPs. Calibration of TWIM based systems to provide absolute collision cross section for proteins is still an issue ^3-5^ and this article does not aim to resolve it. Since no external data is available that would require absolute collisional cross section determination, the focus of this article is mostly on the changes observed in the measured collisional cross-sections. Therefore, calibration was done following the protocol described by Ruotolo et al ^2^. Shvartsburg & Smith ^1^ recommend that a set of multiply charged proteins be used for calibration rather than a single multiply charged protein, covering the expected mass range and cross-section ranges. We chose to use only denatured myoglobin, as is had a similar mass and CCS to that of PrP and not to follow this recommendation, considering that our purpose was mostly a comparison of the changes in cross-sections.

In the resulting plot in Figure S1, one can clearly observe that some charge states seem related to other neighboring charge states forming series of points that we will denote as conformer families. In this denomination, we intend to convey that a single solution-phase conformation is expected to lead to a gas phase charge state distribution with CCS that should be closely related, and with a progression in CCS related to an increase in volume due to increased coulombic repulsion as charge states increase (see for instance ^6^). This term is not totally accurate as one could expect that several conformations might be present in a single conformer family. Conformational landscapes are represented by CCS = f (*z*) plots show that PrPs could encompass more than one family of structures that coexist in the gas phase (See Figure S1). This was confirmed by analysis of mobility graphs of selected CS using MassLynx^TM^ software. To define how significant are changes observed in Ω(z) values, the reproducibility of the experiments and an evaluation of error were performed. To that purpose mobility experiments were repeated 3 times on denatured myoglobin used for calibration and 16 times for ARQ protein prion. For calibration reproducibility, it appears from our experiments that factor *X* calculation was reproducible with an averaged value of 0.602325 and a standard error (σ) of 0.016. From the averages Ω(z) values calculated for the same CS along the sixteen experiments or Ω(z) values calculated for all CS along each conformer family for the sixteen experiments, the standard variation on these two values showed strong reproducibility with a standard deviation (σ) of 11.31 Å^2^. This means that differences of more than 12 Å^2^ for a specific CS that belong to different conformer families should be considered noteworthy. Moreover, this shows that variations of Ω(z) values for each CS inside the same conformer family are reproducible.

***1/ Description of Ovine prion protein constructs***

PrP ARQ PM_av_ 22915.37 Da

SKKRPKPGGGWNTGGSRYPGQGSPGGNRYPPQGGGGWGQPHGGGWGQPHGGGWGQPHGGGWGQPHGGGGWGQGGSHSQWNKPSKPKTNMKHVAGAAAAGAVVGGLGGYMLGSAMSRPLIHFGNDYEDRYYRENMYRYPNQVYYRPVDQYSNQNNFVHDCVNITVKQHTVTTTTKGENFTETDIKIMERVVEQMCITQYQRESQAYYQRGAS

PrP I208M PM_av_ 22933.41 Da

SKKRPKPGGGWNTGGSRYPGQGSPGGNRYPPQGGGGWGQPHGGGWGQPHGGGWGQPHGGGWGQPHGGGGWGQGGSHSQWNKPSKPKTNMKHVAGAAAAGAVVGGLGGYMLGSAMSRPLIHFGNDYEDRYYRENMYRYPNQVYYRPVDQYSNQNNFVHDCVNITVKQHTVTTTTKGENFTETDIK**M**MERVVEQMCITQYQRESQAYYQRGAS

PrP H190A PM_av_ 22849.11 Da

SKKRPKPGGGWNTGGSRYPGQGSPGGNRYPPQGGGGWGQPHGGGWGQPHGGGWGQPHGGGWGQPHGGGGWGQGGSHSQWNKPSKPKTNMKHVAGAAAAGAVVGGLGGYMLGSAMSRPLIHFGNDYEDRYYRENMYRYPNQVYYRPVDQYSNQNNFVHDCVNITVKQ**A**TVTTTTKGENFTETDIKIMERVVEQMCITQYQRESQAYYQRGAS

PrP I206A PM_av_ 22873.09 Da

SKKRPKPGGGWNTGGSRYPGQGSPGGNRYPPQGGGGWGQPHGGGWGQPHGGGWGQPHGGGWGQPHGGGGWGQGGSHSQWNKPSKPKTNMKHVAGAAAAGAVVGGLGGYMLGSAMSRPLIHFGNDYEDRYYRENMYRYPNQVYYRPVDQYSNQNNFVHDCVNITVKQHTVTTTTKGENFTETD**A**KIMERVVEQMCITQYQRESQAYYQRGAS

Construit N-Ter PM_av_ 9960.1 Da

VGLCKKRPKP GGGWNTGGSR YPGQGSPGGN RYPPQGGGGW GQPHGGGWGQ PHGGGWGQPH GGGWGQPHGG GGWGQGGSHS QWN

Construit C-Ter PM_av_ 16147.9 Da

VVGGLGG YMLGS**A**MSRP LIHFGNDYED RYY**R**ENMYRY PNQVYYRPVD **Q**YSNQNNFVH D**C**VNITV**K**QH TVTTTTKGEN FTETDIK**I**ME RVVEQM**C**ITQ YQRESQAYYQ RGAS

**Figure S1:** Collisional cross section (CCS) plot versus charge state (CS, *z*) of ovine prion protein (ARQ) recorded at 10 µM concentration in triethylammonium acetate 10 mM pH 3.3 depicting the conformational landscape (CCS = f (*z*)) of Prion protein. This representation shows charge states presenting overlapping conformers having different drift times and allows grouping by conformer families, as also represented in Table S1. Note that some dots do not refer to ARQ and correspond to noise (low intensity dots).


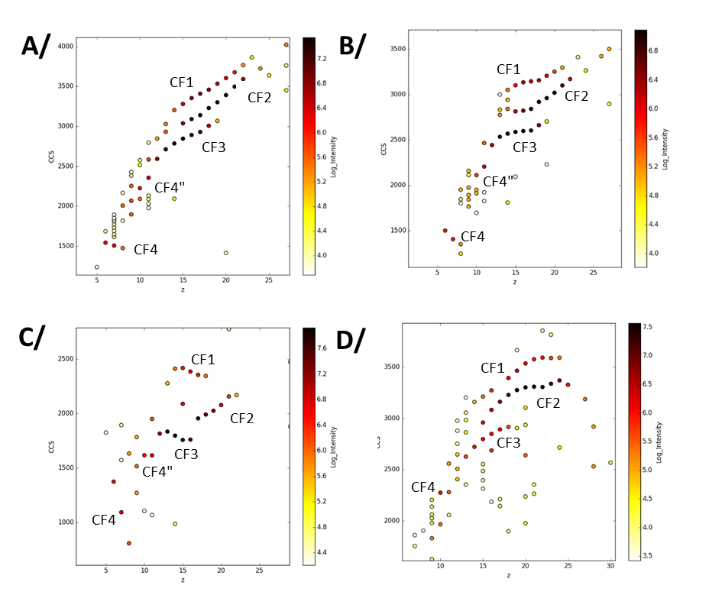


**Table S1:** Drift times in msec observed for each charge state (CS) associated to their respective conformer family (CF) (Lower panel).

**Figure S2:** Mass spectra of ovine ARQ PrP dimer obtained from the beginning, the middle and the end of the SEC chromatographic peak showing the dimer charge state distribution (CSD). Here Prion protein dimer show no significant change in the CSD wherever mass spectra are extracted, since no shift of this last is observed and that peak intensity ratios of the charge state are similar. The only difference observed is due to the presence of some charge states belonging to a contaminant (16033 Da).

**Figure S3:** Plot of the relative intensities of the prion proteins dimer for **A/** ARQ (*wt* PrP); **B/** H190A, **C/** I208M and **D/** I206A mutants in 10 mM triethylammonium acetate pH 3.3 under “native” mode as a function of protein concentration.

**Figure S4:** **A/** Mass spectrum of apo-myoglobin showing the presence of a bimodal charge state distribution (bimodal CSD) suggesting the presence, as previously demonstrated ((1) Wang, F. & Tang, X. Conformational heterogeneity of stability of apomyoglobin studied by hydrogen/deuterium exchange and electrospray ionization mass spectrometry. Biochemistry 35, 4069-4078, (1996). 2) Zaia, J., Annan, R. S. & Biemann, K. The correct molecular weight of myoglobin, a common calibrant for mass spectrometry. Rapid Commun Mass Spectrom 6, 32-36,(1992)). of at least two conformers in the gas phase. **B/** Evolution of intensity ratios of putative myoglobin conformer families CF1 and CF2 with protein concentration showing no significant change in conformer family intensity ratios.

**
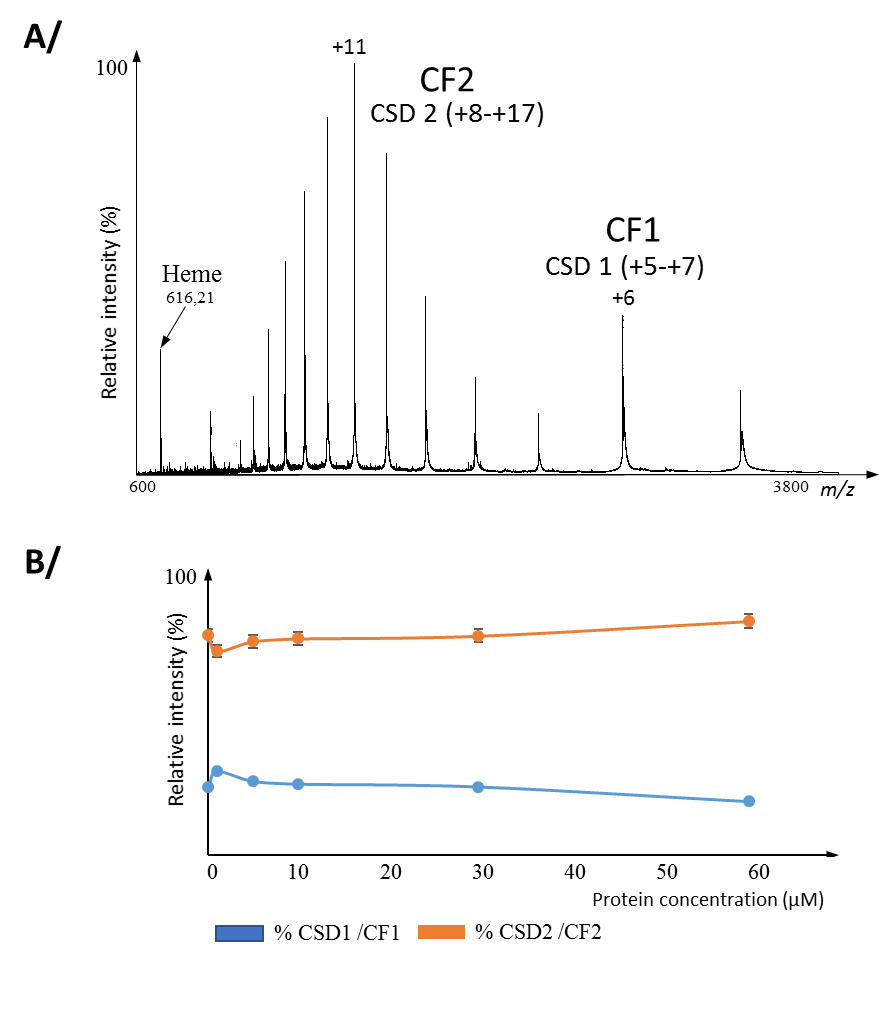
**

**Table S2:** Report of reproducibility by calculating the standard deviations in % for each CF of ARQ and mutants (H190A, I208M and I206A). Reproducibility was calculated for three sample solutions prepared day to day and with which ten ion-mobility / mass spectra were recorded. This protocol was applied for all protein.

**Author information**

* Author for correspondence : Dr F. Halgand, Université Paris Sud- CNRS, Laboratoire de Chimie Physique UMR 8000, Rue Ampère, bâtiment 200 porte 1, 91402, Orsay, France. Email : frederic.halgand@u-psud.fr; Phone : +33 1 69 15 82 51.

**Notes**

The authors declare no competitive financial interest.

**Acknowledgements**

The authors acknowledge the funding by the Ile-de-France DIM Analytics program for the MOBICS project. The authors warmly thank Pr Julian Whitelegge for valuable scientific discussion and Jan Bohl for editing help.

**References**

1 Shvartsburg, A. A. & Smith, R. D. Fundamentals of traveling wave ion mobility spectrometry. *Anal Chem* **80**, 9689-9699, doi:10.1021/ac8016295 (2008).

2 Ruotolo, B. T., Benesch, J. L., Sandercock, A. M., Hyung, S. J. & Robinson, C. V. Ion mobility-mass spectrometry analysis of large protein complexes. *Nat Protoc* **3**, 1139-1152, doi:10.1038/nprot.2008.78 (2008).

3 Bush, M. F., Campuzano, I. D. & Robinson, C. V. Ion mobility mass spectrometry of peptide ions: effects of drift gas and calibration strategies. *Anal Chem* **84**, 7124-7130, doi:10.1021/ac3014498 (2012).

4 Salbo, R. *et al.* Traveling-wave ion mobility mass spectrometry of protein complexes: accurate calibrated collision cross-sections of human insulin oligomers. *Rapid Commun Mass Spectrom* **26**, 1181-1193, doi:10.1002/rcm.6211 (2012).

5 Sun, Y., Vahidi, S., Sowole, M. A. & Konermann, L. Protein Structural Studies by Traveling Wave Ion Mobility Spectrometry: A Critical Look at Electrospray Sources and Calibration Issues. *J Am Soc Mass Spectrom*, doi:10.1007/s13361-015-1244-5 (2015).

6 Beveridge, R. *et al.* A Mass-Spectrometry-Based Framework To Define the Extent of Disorder in Proteins. *Anal Chem* **86**, 10979-10991, doi:10.1021/ac5027435 (2014).
